# Development of a Passive Liquid Valve (PLV) Utilizing a Pressure Equilibrium Phenomenon on the Centrifugal Microfluidic Platform

**DOI:** 10.3390/s150304658

**Published:** 2015-02-25

**Authors:** Wisam Al-Faqheri, Fatimah Ibrahim, Tzer Hwai Gilbert Thio, Norulain Bahari, Hamzah Arof, Hussin A. Rothan, Rohana Yusof, Marc Madou

**Affiliations:** 1 Centre for Innovation in Medical Engineering (CIME), Faculty of Engineering, University of Malaya, 50603 Kuala Lumpur, Malaysia; E-Mails: wisamfakhri83@yahoo.com (W.A.-F.); ainbahari@yahoo.com (N.B.); 2 Department of Biomedical Engineering, Faculty of Engineering, University of Malaya, 50603 Kuala Lumpur, Malaysia; 3 Faculty of Science, Technology, Engineering and Mathematics, INTI International University, Persiaran Perdana BBN, Putra Nilai, 71800 Nilai, Negeri Sembilan, Malaysia; E-Mail: gilbert_thio@hotmail.com; 4 Department of Electrical Engineering, Faculty of Engineering, University of Malaya, 50603 Kuala Lumpur, Malaysia; E-Mail: ahamzah@um.edu.my; 5 Department of Biomedical Engineering, University of California, Irvine, 92697 CA, USA; E-Mail: mmadou@uci.edu; 6 Department of Mechanical and Aerospace Engineering, University of California, Irvine, 92697 CA, USA; 7 Department of Molecular Medicine, Faculty of Medicine, University of Malaya, 50603 Kuala Lumpur, Malaysia; E-Mails: rothan@um.edu.my (H.A.R.); rohana@um.edu.my (R.Y.)

**Keywords:** centrifugal platform, microfluidic CD, passive liquid valve, pressure equilibrium, S-PLV, D-PLV

## Abstract

In this paper, we propose an easy-to-implement passive liquid valve (PLV) for the microfluidic compact-disc (CD). This valve can be implemented by introducing venting chambers to control the air flow of the source and destination chambers. The PLV mechanism is based on equalizing the main forces acting on the microfluidic CD (*i.e.*, the centrifugal and capillary forces) to control the burst frequency of the source chamber liquid. For a better understanding of the physics behind the proposed PLV, an analytical model is described. Moreover, three parameters that control the effectiveness of the proposed valve, *i.e.*, the liquid height, liquid density, and venting chamber position with respect to the CD center, are tested experimentally. To demonstrate the ability of the proposed PLV valve, microfluidic liquid switching and liquid metering are performed. In addition, a Bradford assay is performed to measure the protein concentration and evaluated in comparison to the benchtop procedure. The result shows that the proposed valve can be implemented in any microfluidic process that requires simplicity and accuracy. Moreover, the developed valve increases the flexibility of the centrifugal CD platform for passive control of the liquid flow without the need for an external force or trigger.

## 1. Introduction

Compared with conventional diagnostic methods, microscale fluidic platforms have attracted increasing interest due to the dramatic decrease in sample/reagent volumes and time which are just a few of the many advantages of these platforms [1]. In the last few decades, many microfluidic platforms have been reported and tested as diagnostic methods for different diseases. Most of the reported microfluidic platforms fall under two main categories: stationary platforms (e.g., Lab-on-Chip (LOC)) and spinning platform (e.g., Lab-on-Disc (LOD), also known as a microfluidic CD) [1,2,3]. On the microfluidic CD platform, two main forces affect the liquid flow, namely, centrifugal pressure and capillary pressure. The centrifugal pressure is a force that depends on the spinning speed and pushes the liquid towards the outer edge of the platform. In contrast, the capillary pressure is independent of the spinning speed and acts against liquid flow. At a specific spinning speed, the centrifugal pressure overcomes the capillary pressure, causing the liquid to flow, and this spinning speed is called the burst frequency. Therefore, the centrifugal pressure and capillary pressure can be utilized to pump and control, respectively, the liquid flow in this platform. However, with the implementation of more complex multi-stepped processes, the use of only centrifugal and capillary pressure to control liquid flow has become a real challenge in this field. Several multi-stepped biomedical processes were successfully implemented on the LOD platform, and some example applications include enzyme linked-immunosorbent assays (ELISAs) [4,5,6,7], real time polymerase chain reaction (PCR) [8,9], and plasma or particle separation [10,11,12].

Recently, different valving methods to control fluid flow in microfluidic platforms (LOC & LOD) were reported. Passive and active valves are the two major categories for the newly presented microvalving methods [1,2]. Various examples for passive valves include hydrophobic and hydrophilic valves [13], flap valves [14], siphon valves [15], passive check valves [16,17], and gating valves [18]. Furthermore, different active valving methods have been reported, such as wax valves [19,20,21], ice valves [8], polymer optofluidic valves [22], hydrogel valves [23] and active check valves [24,25]. Active values require an external actuation force, which is one of the main disadvantages of these valves compared with passive valves.

In our previous work, we presented an active wax valve to control the burst frequency by applying a vacuum and/or compression effect on the source and destination chamber respectively [19]. In this work, we propose an easy-to-utilize passive valving method that employs the same mechanism of controlling the air flow of the source and destination chambers; however, instead of using paraffin wax, a venting chamber acting as a liquid valve controls the venting holes of both chambers. In contrast with the previous valve, the developed liquid passive valve (PLV) is a multi-actuation, easy-to-implement, and highly controllable valve. The developed valving method is based on the idea of equalizing the pressure that two separate volumes of liquids generate on the microfluidic CD (*i.e.*, the source chamber liquid, and the venting chamber liquid). Three different parameters that affect the performance of the developed PLV (*i.e.*, liquid height in the venting chamber, liquid density, and venting chamber distance from the CD center) are carefully tested, and the results are reported and compared with the theoretical results. For a better demonstration of the proposed valve, microfluidic liquid switching and liquid metering processes were performed. Moreover, Bradford assay for measuring protein concentration was conducted using the new valve. The results show that the burst frequency of the presented PLV can be increased by slightly adjusting the height and density of the liquid in the venting chamber. Moreover, the burst frequency can also be adjusted by changing the position of the venting chamber on the microfluidic CD. Finally, the proposed valve can be utilized in a wide range of multistep processes that require simplicity, biocompatibility and portability.

## 2. Materials and Methods 

In this section, the microfluidic design and the operational mechanism of the developed PLV is discussed in detail. First, the PLV design and the microfluidic CD fabrication method are explored. Then, the operation mechanism is discussed based on the controlled chamber: a passive liquid valve to control the source chamber (S-PLV) and a passive liquid valve to control the destination chamber (D-PLV). An analytical model is described to understand the physical forces involved in the operation of the developed valve. Finally, the experimental setup of the proposed valve is presented in the last section. For better understanding of the different microfluidic designs and the fabricated CD layers, please refer to the supplementary material included with this paper.

### 2.1. Design and Fabrication 

**Figure 1:**
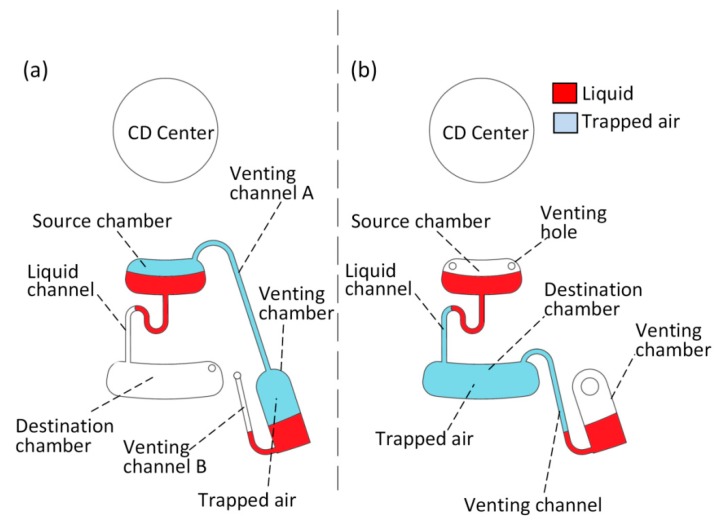
PLV designs, (**a**) S-PLV design where the venting chamber is connected to the source chamber through the venting channel, (**b**) D-PLV design where the venting chamber is connected to the ventless destination chamber through the venting channel.

In this study, two microfluidic designs were fabricated to experimentally test the developed valve. Figure 1 shows the designs of an S-PLV (Figure 1a) and a D-PLV (Figure 1b). Both designs consist of three chambers: a source chamber (1 mm deep), a destination chamber (1 mm deep), and a venting chamber (2.5 mm deep). The three chambers are connected together by liquid and venting channels that are 0.7 mm wide and 0.5 mm high. For the S-PLV design (see Figure 1a), the venting chamber is connected to a ventless source chamber via venting channel A. With this design and the trapped air, the flow of the liquid in the source chamber is controlled by the liquid in the venting chamber. In contrast, in the D-PLV design (see Figure 1b), the venting chamber is connected to a ventless destination chamber. Therefore, the air flow of the ventless destination chamber is controlled by the liquid in the venting chamber. The proposed valves depend on trapped air, and air compresses and expands easily; thus, the used of straight channels to connect the source and destination chambers generates fluctuations in the valving performance. This fluctuation is caused by the momentum of the liquid as it bursts from the source chamber, and this momentum can lead to overstretching of the air (in the case of S-PLV) or overcompression of the air (in the case of D-PLV) and thus prevents proper operation of the valve. To overcome this limitation, a U-shaped bent was introduced in the micro-channel that connects the source and destination chambers to reduce the liquid flow speed and the associated turbulence caused by the liquid momentum (see Figure 1a and 1b). This U-shaped channel reduces the liquid flow speed and thus, the trapped air produces a more gradual negative pressure (in the case of S-PLV) or gradual positive pressure (in the case of D-PLV).

The microfluidic CD fabricated for this study consists of three layers: two polymethyl methacrylate (PMMA) layers and one pressure-sensitive adhesive (PSA) layer. The bottom 4 mm thick PMMA layer contains all the microfluidic chambers and channels. The top 2 mm thick PMMA layer contains the venting holes which also serve as liquid loading holes and as alignment holes. The adhesive PSA layer bonds the two PMMA layers to each other.

### 2.2. Operation Mechanism 

To understand the operation mechanism of the developed valve, we must first define the fundamental pressures acting on a fully passive microfluidic CD (without PLV). On a CD, any liquid loaded into a chamber experiences a centrifugal pressure when the CD is spun. This centrifugal pressure pushes the liquid away from the center axis of the spinning platform, and it can be calculated using the following equation [1,26]:

(1) $$P_{centrifugal}=\rho \omega^{2}\Delta r\bar{r}$$

where *ρ* is the density of the liquid, *ω* is the rotational speed of the CD in radians per second (rads−1), Δ*r* is the difference between the top and bottom of the liquid levels at rest with respect to the center of the CD, and $\bar{r}$ is the average distance of the liquid from the CD center. In contrast, the capillary pressure within a micro-channel acts against the centrifugal pressure, and it prevents the liquid from flowing forward into the channel. That capillary pressure can be calculated using the following equation [26]:

(2) $$P_{cap}=\frac{4\cos\theta_{c}\gamma_{la}}{D_{h}}$$

where *θ_c_* is the liquid contact angle, *γ_la_* is the liquid-air surface energy, and *D_h_* is the channel hydraulic diameter. 

**Figure 2:**
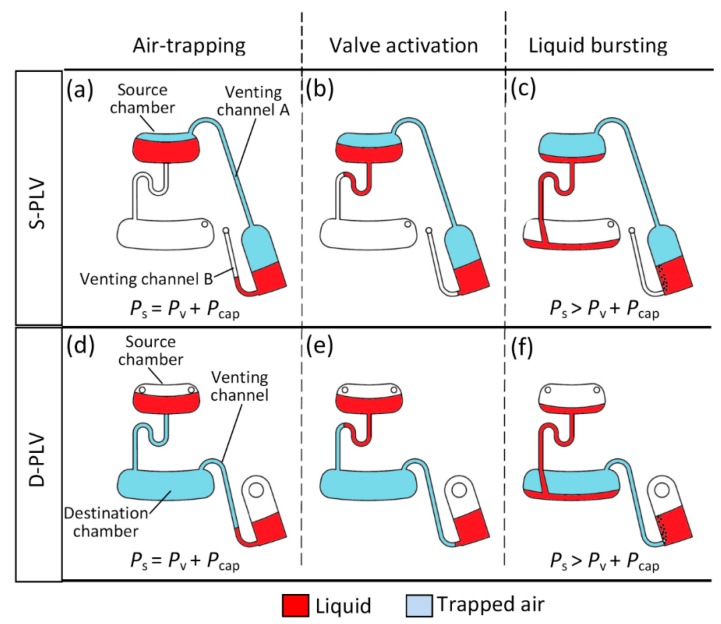
Liquid valving sequence, (**a**) S-PLV at low frequency; (**b**) high spinning speed where the source chamber liquid starts to flow inside the micro-channel, creating a lower pressure effect in the venting chamber; (**c**) high frequency, the source chamber liquid bursts into the destination chamber; (**d**) D-PLV at low frequency; (**e**) high speed, the source chamber liquid starts flowing into the microchannel, creating compression state in the destination chamber; (**f**) high frequency, the source chamber liquid bursts into the destination chamber.

Now, the PLV mechanism can be broken into three main operational stages: air trapping, valve actuation, and liquid bursting. Figure 2a–c show the liquid in the three different stages for the S-PLV, and Figure 2d–f show the operational stages for the D-PLV. During the three operational stages, the various pressures involved are the centrifugal pressure acting on the liquid in the source chamber, *P_s_*, the centrifugal pressure acting on the liquid in the venting chamber, *P_v_*, and the capillary pressure against the liquid in the source chamber, *P_cap_*. The *P_s_* pressure constantly pushes the source chamber liquid towards the outer edge of the CD, while *P_v_* and *P_cap_* both act against liquid flow. Note that *P_s_* and *P_v_* are the result of centrifugal pressure on the microfluidic CD. These pressures are calculated using the fundamental centrifugal pressure equation (Equation (1)). As mentioned before in subsection “*2.2. Operation mechanism*”, this is true for any liquid loaded onto the centrifugal microfluidic CD. However, the calculation needs to be made with respect to the parameters related to the source chamber and the venting chamber such as the liquid density and the distance from the center of rotation. 

*Air Trapping Stage*: the testing of the S-PLV starts with an injection of 40 µl of the colored DI water into the source chamber. Afterward, the required amount of liquid (for testing the effect of the liquid height on the burst frequency) or the required type of liquid (for testing the effect density on the burst frequency) is injected into the venting chamber. Then, the preloaded CD is mounted on the spin test system, and the spin speed is gradually increased. Initially, the pressures in the system are at equilibrium, and some air is trapped between the source and venting chambers (see Figure 2a). The critical point just prior to liquid bursting from the source chamber is achieved when the pressures in the system are balanced as follows:

(3) $$P_{s}=P_{v}+P_{cap}$$

To determine whether the valve holds liquid back in a source chamber or whether the liquid bursts from the source chamber, a balance pressure, *P_balance_*, which is the difference between the pressures is determined as follows:

(4) $$P_{balance}=P_{s}-(P_{v}+P_{cap})$$

**Figure 3:**
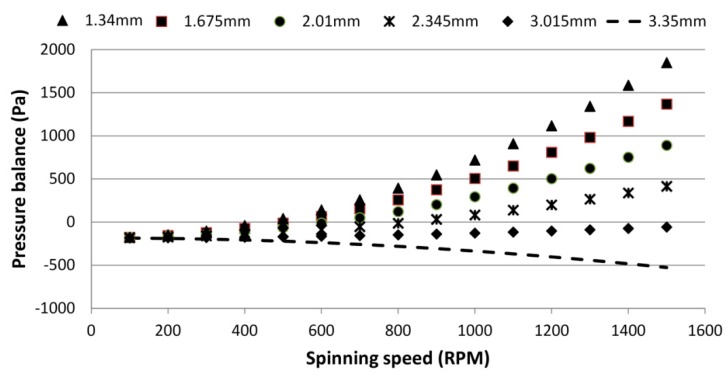
Pressure balance at different venting chamber liquid heights for a range of spinning speeds.

A positive *P_balance_* indicates that liquid will burst from the source chamber, whereas a negative P*_balance_* means that the PLV will hold the liquid back in the source chamber. Figure 3 shows the range of *P_balance_* for different liquid heights in the venting chamber. The result in Figure 3 is valid for both S-PLV and D-PLV as both have the same fundamental principle of operation (based on the centrifugal force *vs.* capillary pressure and venting chamber pressure). At low frequencies, all venting chamber heights produce a negative balance pressure, indicating that the PLV does hold the liquid back. When the spinning speed is increased, the *P_s_* pressure starts to increase and overcomes the sum of *P_v_* and *P_cap_* (giving positive *P_balance_* values). However, when the liquid height in the venting chamber is higher than a critical value (such as 3.015 mm and 3.35 mm) where the combination of the liquid height in the venting chamber and the position in respect the center of the rotation produce *P_v_* value that always higher than *P_s_*, *P_balance_* will always be negative. In other words, the valve will hold the liquid in the source chamber indefinitely. Table 1 presents all the parameters implemented in the analytical calculations of *P_balance_*. 

**Table 1:** Values of the parameters implemented in the analytical calculations.

Parameters	Values	Parameters	Values	Parameters	Values
*Ɵ_c_*	68°	*r_s1_*	32.3 mm	*r_v2_*	60 mm
*γ_la_*	71.97 mN/m	*r_s2_*	37.5 mm	*ρ*	1.000 g/cm^3^
*D_h_*	3.83e^−4^ m	*r_v1_*	58.66, 58.32, 58, 57.65, 56.98 and 56.65 mm		

*Valve Actuation Stage*: when the spinning speed increases, some liquid from the source chamber starts flowing into the microchannel towards the destination chamber (see Figure 2b). The resulting decrease in liquid level in the source chamber will momentarily expand the volume of trapped air on top of the source and venting chambers. This air expansion creates a state with an instantaneous negative pressure lower than the atmospheric pressure outside the system. This pressure change acts as an actuator that allows the liquid in the venting chamber to flow in the direction of the liquid flow of the source chamber. This negative air pressure tries to suck air through the liquid in the venting chamber. At the same time, the centrifugal pressure acting on the liquid in the venting chamber acts against this air-pulling process. Thus, the valve is actuated, and the burst frequency of the source chamber liquid will increase as long as the venting chamber liquid prevents air from flowing into the system through venting chamber B. Note that the air expansion occurs in this stage is not included in the developed model as it describes the instances of liquid bursting. At the instance of liquid bursting, air expansion and compression is instantaneous, low, and negligible.

*Liquid Bursting Stage*: as shown in Figure 2b, as the spinning speed further increases, the increased centrifugal pressure on the source chamber liquid (*P_s_*) tries to suck air through the liquid in the venting chamber by first emptying venting channel B. Further increasing the spinning speed then causes the liquid in the source chamber to approach the top of the U-shaped bent and the pressure acting on the source chamber liquid, *P_s_*, eventually overcomes the combination of the venting chamber pressure (*P_v_*) and the channel capillary pressure (*P_cap_*). At this point, air is forced to enter the system through the venting chamber (bubbles are observed in the venting chamber liquid), and the source chamber liquid is fully transferred to the destination chamber (see Figure 2c). 

The principle of operation of the D-PLV is similar to that of the S-PLV; however, instead of using trapped air on top of the source chamber, the D-PLV works by compressing the air in the destination chamber (see Figure 2d). Figure 2d–f shows the air-trapping stage, valve actuation stage and liquid bursting stages, respectively, for the D-PLV valve. 

In this study, we are also interested in calculating the burst frequency (*Bfreq_PLV_*) of the liquid in the source chamber when any of the relevant parameters (*i.e.*, venting chamber liquid height, liquid density, and venting chamber position) are changed. Using Equation (1) to expand *P_s_* and *P_v_* in Equation (3), we obtain the following expression:

(5) $$\rho_{s}\omega^{2}\Delta r_{s}\bar{r_{s}}=\rho_{V}\omega^{2}\Delta r_{V}\bar{r_{V}}+P_{cap}$$

Further rearranging equation (5) then yields:

(6) $$Bfreq_{PLV}=\sqrt{\frac{P_{cap}}{\rho_{s}\Delta r_{s}\bar{r_{s}}-\rho_{V}\Delta r_{V}\bar{r_{V}}}}\left(\frac{30}{\pi }\right)$$

Using Equation (6), the effect of various parameters that control the effectiveness of the proposed valve can be studied theoretically. In this study, three different parameters: (i) the liquid height of the venting chamber, (ii) the density of the liquid in the venting chamber, and (iii) the venting chamber distance from the CD center, are evaluated experimentally.

### 2.3. Experimental Setup 

To fabricate the designed microfluidic CDs, PMMA plastic and PSA adhesive are utilized as the building materials for the platform. The designed micro-features are milled in 4 mm thick PMMA transparent plastic discs (from Asia Ply Industrial Sdn. Bhd., Selangor, Malaysia). To perform the micro-milling process, a computer numerical control (CNC) machine (model VISION 2525, by Vision Engraving and Routing Systems, Phoenix, AZ, USA) is used. A pressure-sensitive adhesive (PSA) material (by FLEXcon, Spencer, MA, USA) is applied in a thin layer between the top and bottom PMMA layers for bonding purpose. Micro-features in the PSA material are cut using a cutter plotter (model PUMA II, by GCC, New Taipei, Taiwan). After the features are cut in all the layers of the CD, a custom-made press roller is used to generate tight binding. Coomassie Brilliant Blue G-250 dye and bovine serum albumin (BSA) are used to perform the Bradford assay on the CD. A BIO RAD 680 colorimetric micrewell plate reader is utilized to read the assay results. The spinning process is performed using a custom-made spinning CD system that is equipped with a special high speed camera.

## 3. Microfluidic Applications Using PLV 

**Figure 4:**
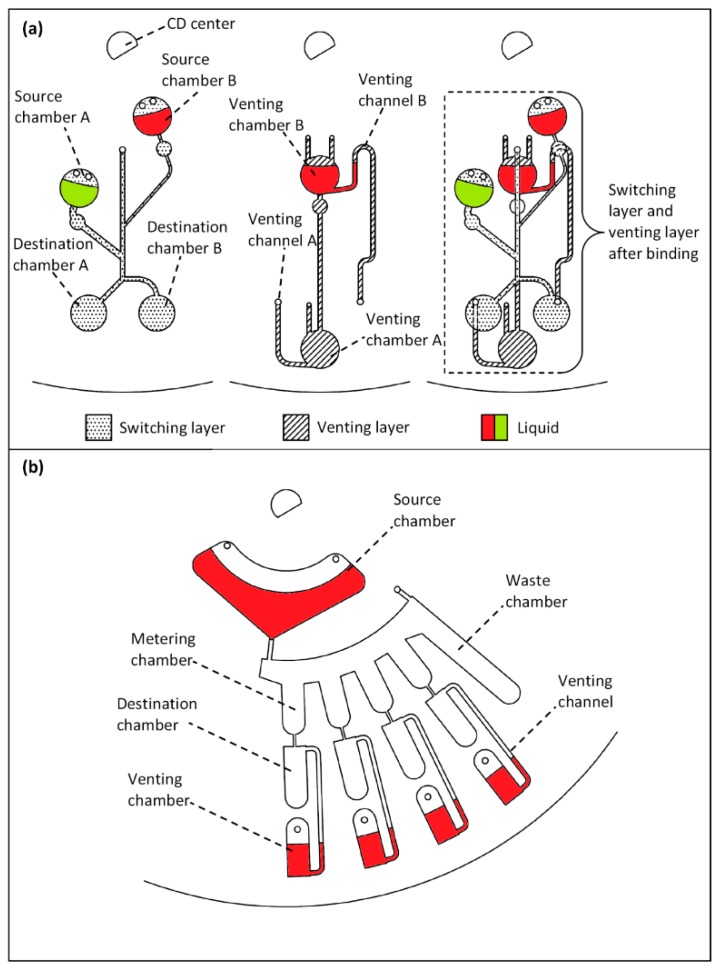
Microfluidic CD design for liquid switching and liquid metering (**a**) layer-by-layer design of the liquid switching process (**b**) design of the liquid metering process.

Various microfluidic processes can be performed utilizing the proposed PLV valves. In this paper, microfluidic liquid switching and liquid metering are conducted using the D-PLV valving method. Figure 4a and 4b show the microfluidic CD designs of the liquid switching and metering processes, respectively.

The liquid switching design consists of two main layers: the switching layer and the venting layer. The switching layer contains two source chambers (A & B) connected to two destination chambers (A & B). The source chambers are placed at different distances from the CD center and thus burst at different rotational speeds (see Figure 4a). Furthermore, the venting layer consists of two venting chamber (A & B) that are connected to two venting channels (A & B). For liquid switching, the venting channels of the two destination chambers are controlled using two venting chambers (A & B). For a more practical use of the CD space, the design features are engraved on two different layers that appear to overlap when viewed from the top (3D CD design). This design is fabricated using a five layer CD (please refer to the supplementary material). To clearly observe the switching process, two solutions of colored DI water (red and green) are utilized in this process.

The liquid switching experiment starts with the injection of 20 µL of green DI water into source chamber A with the injection of the same volume of the red DI water into source chamber B. The second step is the injection of 60 µL of red DI water into venting chamber B. This process will create a normally opened valve for destination chamber A and a normally closed valve for destination chamber B. Afterwards, the CD is mounted on the spinning CD system, and the spinning process starts. The switching process can be divided into three main steps. In Step 1, the liquid of source chamber A bursts and is forced to flow towards destination chamber A as the venting channel of destination chamber B is closed by venting chamber B. In Step 2, the liquid in venting chamber B bursts into venting chamber A; this burst opens the venting hole of destination chamber B while sealing venting channel A. In Step 3, liquid bursting from source chamber B will then flow towards destination chamber B without entering destination chamber A. 

Figure 4b shows the design for the liquid metering. The design consists of a main source chamber that contains the liquid that will be metered. The source chamber is connected to four metering chambers (90 µL volume each) and one waste chamber; the excess liquid will overflow into the waste chamber. Finally, the four metering chambers are connected to ventless destination chambers, which are controlled by four separate venting chambers. The metering process starts with the injection of 550 µL of the red DI liquid into the source chamber. Then, 50 µL of the same DI water is injected into each venting chamber. Afterward, the microfluidic CD is mounted on the CD spinning system, and the CD is spun. With this simple design, the 550 µL of DI water can be automatically metered (90 µL) and then transferred into the four destination chambers for use in the next stage in a multistep CD.

## 4. Results and Discussion

In this section, the experimental results of the proposed valving mechanism are presented. Moreover, the experimental results are compared with the theoretical calculations to assess the analytical model we described earlier. For both the S-PLV and D-PLV designs, the control burst frequency (when there is no liquid in the venting chamber) was tested to be 275 RPM. The U-shaped bent channel increases the burst frequency by 10 RPM because the control burst frequency in the case of a straight microchannel was tested to be 265 RPM. This 10 RPM difference results from the short distance of the reverse flow in the second leg of the U-shaped bent. Each experiment in this section was repeated10 times for validation of the results.

### 4.1. Effect of the Liquid Height on the Burst Frequency

**Figure 5:**
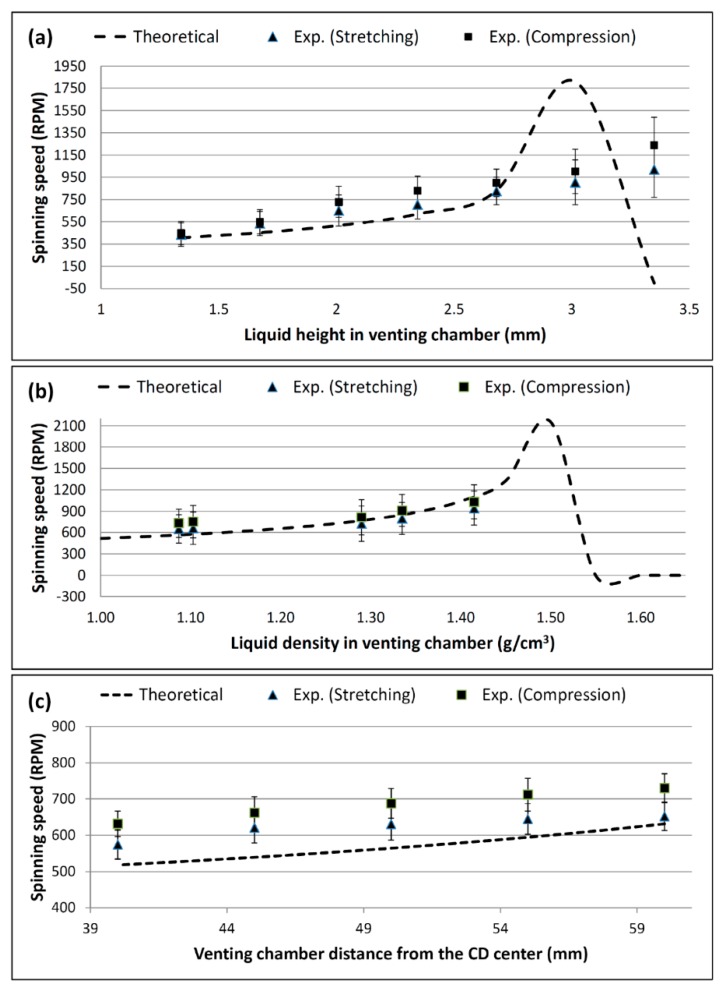
Theoretical and experimental results for (**a**) the effect of different venting chamber liquid heights on burst frequency, (**b**) the effect of different venting chamber liquid densities on burst frequency, and (**c**) effect of different venting chamber positions on burst frequency (theoretical result calculated using Equation (6)).

To test the effect of the venting chamber liquid height on the burst frequency, a range of liquid heights from 1.34 to 3.35 mm of deionized colored water (corresponding to 20 to 50 µL) was loaded into a venting chamber. Figure 5a shows the experimental results demonstrating the effect of liquid height on the burst frequency for both the S-PLV and D-PLV valves. Moreover, the theoretical result using Equation (6) is included in the figure for comparison purposes. 

From Equation (1), it is obvious that the height of the liquid in the venting chamber will affect the centrifugal pressure acting on the liquid in the venting chamber, and this effect in turn will affect the burst pressure (frequency). From the design of the venting chamber in this study, each additional 1 µL of liquid is equivalent to a 0.067 mm increase in the liquid height in the venting chamber. As an example, 50 µL of liquid in the venting chamber produces an effective liquid height of approximately 3.35 mm.

The theoretical result in Figure 5a shows that initially the burst frequency gradually increases as the venting chamber liquid height increases. Then, a sudden steep increase in the burst frequency for liquid heights greater than 2.68 mm is followed by a rapid drop in the burst frequency (after 3.015 mm). The experimental results show good agreement with the theoretical result for chamber liquid heights in the range between 1.34 mm to 2.68 mm. For both S-PLV and D-PLV, it is clear that the burst frequency is almost doubled from 275 RPM (for the control without liquid in the venting chamber) to 405 RPM by an increase of only 1.34 mm in the liquid height in the venting chamber. 

For a chamber liquid height of 3.015 mm (exceeding 2.68 mm), the liquid height in the venting chamber starts to be close or equal to the liquid height in the source chamber. Therefore, the theoretical result curve peaks at the highest burst frequency and then drops to zero (no bursting) when the liquid pressure of the venting chamber is always higher than the pressure of the source chamber (see Figure 5a). However, the experimental result does not follow the theoretical result because air, which is slightly elastic, can only withstand a limited amount of stretching/compression. If a high pressure is suddenly applied on the trapped air (such as when venting chamber liquid height is more than 3.015), it will be overstretched/overcompressed and the valve will not work or will not follow the theoretical calculations. This air effect and elasticity limitation is well described by Soroori *et al.* [27]. Therefore, in Figure 5, when the spinning speed exceeds the specific threshold, the source chamber will burst although the theoretical calculation says otherwise.

### 4.2. Effect of Liquid Density on the Burst Frequency

In this experiment, the effect of the density of the liquid in the venting chamber on the burst frequency is investigated. The experimental and theoretical results for five different liquids with five different densities are presented in Figure 5b. The following five liquids were used: distilled water (1.000 g/cm^3^), soy milk (1.103 g/cm^3^), glycerol (1.290 g/cm^3^), purified juice (1.335 g/cm^3^), and honey (1.415 g/cm^3^). The liquid densities were measured using a DMA 4100 Density Meter (U-Tube, Anton Paar, Wundschuh, Austria). In each experiment, 30 µL of each liquid was injected into the venting chamber while the source chamber was loaded with 40 µL of distilled water. 

Figure 5b shows that an increase in the liquid density in the venting chamber from 1.000 g/cm^3^ to 1.450 g/cm^3^ increases the burst frequency from approximately 500 RPM to 1200 RPM. In this range, the experimental result shows good agreement with the theoretical calculation. In a manner similar to the effect of the chamber liquid height, when the venting chamber density exceeded 1.450 g/cm^3^, the theoretical result shows a sharp increase where the burst frequency exceeds 2100 RPM. Then, the burst frequency dropped to zero, which corresponds to the valve holding the liquid in the source chamber indefinitely.

### 4.3. Effect of the Venting Chamber Position on the Burst Frequency

In this part of the study, the effect of changing the position of the venting chamber relative to the CD center is investigated. The position of the venting chamber was varied from 40 mm to 60 mm from the CD center in steps of 5 mm. In each experiment, the venting chamber was loaded with 30 µL of distilled water while the source chamber was loaded with 40 µL. 

Figure 5c shows the experimental and theoretical results of this study. Increasing the relative distance between the venting chamber and the CD center steadily increases the burst frequency of the source chamber liquid. When the distance from the CD center is increased from 40 mm to 60 mm, the burst frequency increases by approximately 100 RPM (from 520 to 620 RPM). Although the S-PLV valve yielded experimental results that were closer to the theoretical calculations, the D-PLV valve was more effective in increasing burst frequency.

### 4.4. Liquid Switching and Liquid Metering Results

**Figure 6:**
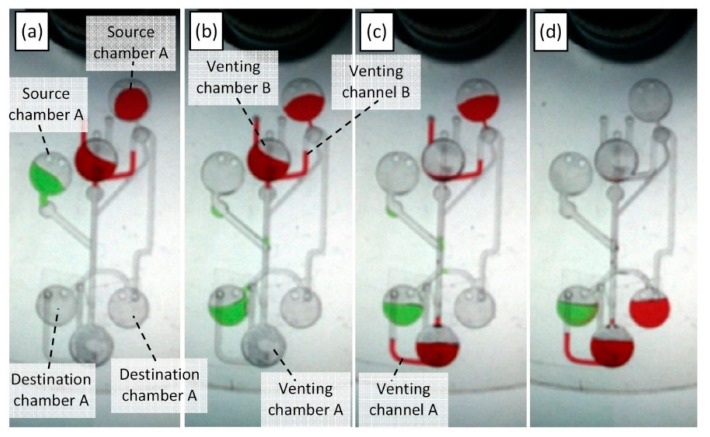
Photos of the microfluidic switching process at various stages (**a**) spinning speed less than 250 RPM where all liquids at its original positions, (**b**) source chamber A liquid bursts towards destination chamber A at 260 RPM, (**c**) venting chamber B liquid bursts towards venting chamber A at 300 RPM, and (**d**) source chamber B liquid bursts towards destination chamber B at 370 RPM.

**Figure 7:**
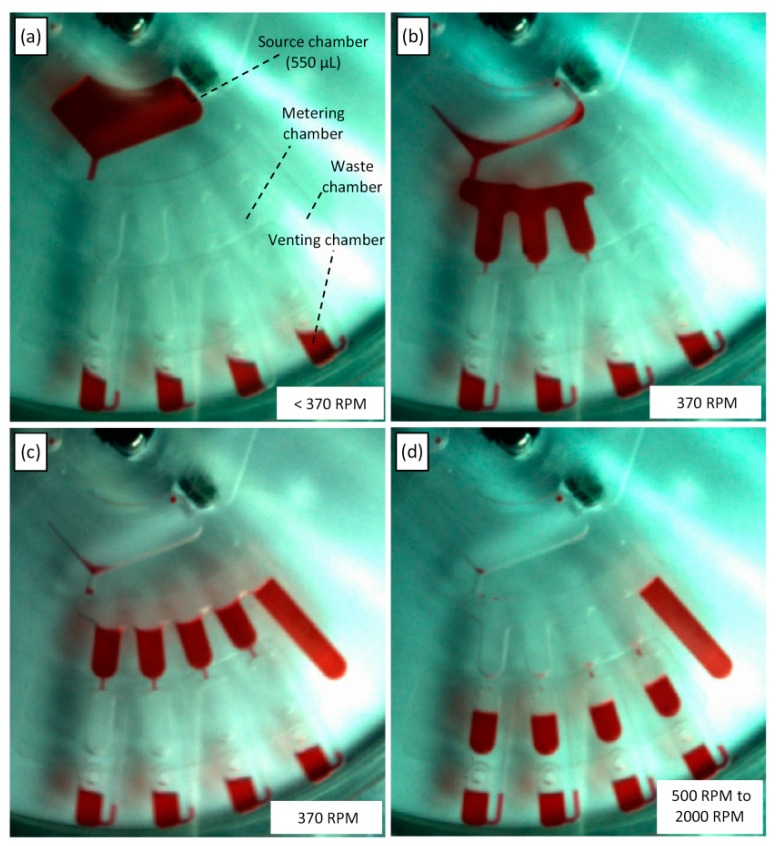
Photos of liquid metering at various stages of the process, (**a**) spinning speed less than 370 RPM, (**b**) & (**c**) the metering chambers are filled whereas the extra liquid overflowed to the waste chamber at 370 RPM, and (**d**) liquid transferred from the metering chamber towards the destination chambers (500 RPM to 2000 RPM).

Photos of the microfluidic liquid switching and liquid metering processes at various stages of the experiment are presented in Figure 6 and Figure 7, respectively. Figure 6a shows the microfluidic CD at the preliminary stage with a spinning speed lower than 250 RPM where all liquids are in their original positions. When the CD spinning speed is increased to 260 RPM, the green liquid in source chamber A bursts (see Figure 6b). The liquid flow is forced towards destination chamber A by the effect of the PLV valve on venting chamber B. Then, the spinning speed is increased to 300 RPM, and the liquid in venting chamber B to burst towards venting chamber A (see Figure 6c). This step will release the venting channel of destination chamber B while sealing the venting channel of destination chamber A. Therefore, when the liquid in source chamber B bursts at 370 RPM, the flow is forced towards destination chamber B (see Figure 6d). 

Two observations were made during our experiments: (1) Some residual liquid can stay in venting channel B after the liquid burst from venting chamber B (see Figure 6c). This liquid will not affect the switching process because the liquid volume is very low (low pressure), and this liquid will be pushed back as soon as the liquid in the source chamber B bursts towards destination chamber B. (2) Often, a drop of the liquid in source chamber B will flow towards destination chamber A due to the high speed bursting, which overcompresses the trapped air in destination chamber A. 

Compared with the switching process proposed previously by Kazemzadeh *et al*. [18], the present liquid switching process shows two main advantages: first, the process can be performed at a lower spinning speed (less than 400 RPM), and this speed is adjustable depending on the utilized venting chamber specifications. Moreover, compared with the wax valve switching proposed by Al-Faqheri *et al.* [19], the PLV valve can be easily and passively performed without any need for an external force or trigger. Finally, the valve state of the proposed PLV can be reversed from normally opened to normally closed and *vice versa*. This capability is a very important feature for some applications where the valve must be switched *ON* and *OFF* at different stages of the process.

Figure 7a shows the metering process at a low spinning speed. When the spinning speed is increased to 370 RPM, the source chamber liquid bursts and fills the metering chambers (90 µL each), whereas the extra liquid overflows to the waste chamber (see Figure 7b and 7c). Finally, the spinning speed increased steadily to 500 RPM, when the liquid in the metering chambers starts to burst to the destination chambers. However, the spinning speed must be steadily increased to 2000 RPM for all the liquid to be transferred to the destination chambers. The reason for the speed increase is as follows: as liquid flows away from the metering chamber, the decreasing liquid height results in a reduction in liquid pressure. Therefore, the spinning speed is increased to increase the pressure on the metering chamber liquid to maintain the bursting of liquid. 

This experiment shows that the proposed PLV valve is capable of controlling liquid that flows at a high speed. In addition, multistep process can be performed on the microfluidic CD utilizing the developed valve. Compared with the pneumatic metering process proposed by Mark, *et al.* [28], the process that is controlled using PLV liquid is more controllable/adjustable because the spinning speed can be increased or decreased depending on user requirements. Finally, compared with the metering method proposed by Al-Faqheri *et al.* [19], this valve is easier to implement and will extend the flexibility of microfluidic CDs by allowing for passive control of fluid flow without the need for active valving.

### 4.5. Bradford Assay for Measuring Protein Concentrations

As a proof of concept, a Bradford assay for measuring protein concentrations is performed utilizing the proposed PLV valve. The Bradford procedure is a well-known colorimetric assay that utilizes the color shift of the utilized reagent (Coomassie Brilliant Blue G-250) to calculate the protein concentration in a specific sample [29]. When the Bradford reagent, which is originally brown, is mixed with a specific sample, the mixture will become blue. The intensity of the blue corresponds to the protein concentration in that specific sample. 

**Figure 8:**
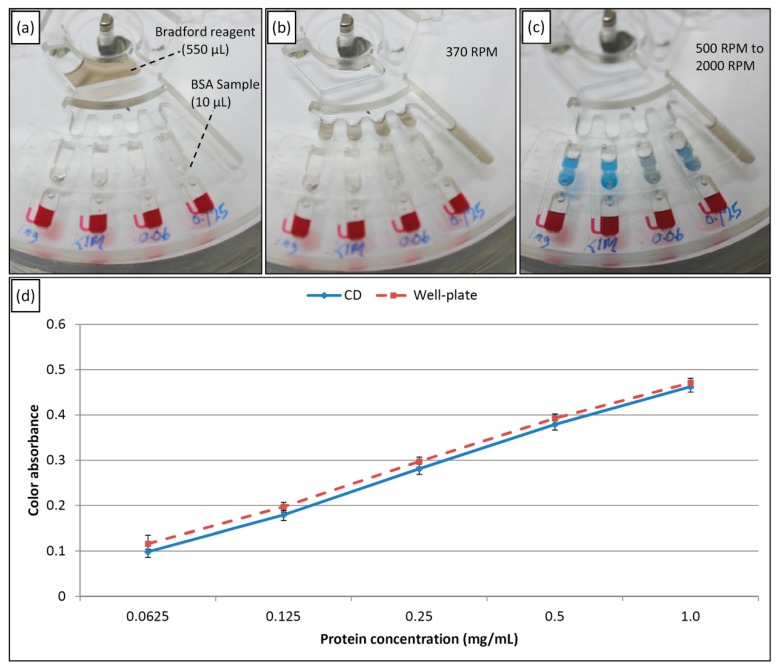
Experimental result of a Bradford assay to measure the protein concentration, (**a**) spinning speed less than 370 RPM, with the Bradford reagent in the source chamber and the samples in the destination chambers, (**b**) The Bradford reagent bursts and fill the metering chambers at 370 RPM, (**c**) the Bradford reagent bursts from the metering chamber to the destination chambers to mix with the BSA samples (500 RPM to 2000 RPM), and (**d**) results for different BSA samples with different concentrations using the microfluidic CD and the micro-well plate as assay platform.

To perform the Bradford assay, the liquid metering design is utilized to test various samples in parallel (see Figure 8a). A BSA sample with a known protein concentration of 10 mg/mL is used in this assay. The BSA is serially diluted five times to obtain samples with protein concentrations of 1, 0.5, 0.25, 0.125, and 0.0625 mg/mL. 

As shown in Figure 8a, the experiment starts with the injection of 550 µL of the Bradford reagent into the source chamber and the injection of 10 µL of each prepared concentration into the destination chambers. Then, the inlet and venting holes of the preloaded destination chambers are tightly sealed using a PSA product. Afterward, 50 µL of red DI water is injected into each venting chamber. Next, the microfluidic CD is mounted on the CD spinning system, and the rotation process is started. By the same steps as those described for the previous metering experiment, the source chamber liquid (with Bradford reagent) is burst at 370 RPM to fill each of the metering chambers with 90 µL of Bradford reagent (see Figure 8b). Then, at increasing speeds of 500 to 2000 RPM, the metered Bradford reagent bursts to the destination chambers to be mixed with the preloaded BSA samples. To ensure good mixing between the reagent and the BSA, a multistep process comprising a batch mode (stop flow) mixing method and manual pipette mixing is performed [30]. Figure 8c shows that the resulting mixture has different intensities in the blue color region depending on the protein concentration in the sample. Finally, after the mixtures are incubated for 5 min at room temperature, the mixtures are pipetted into a 96 micro-well plate and read at 450 nm. To evaluate the present method, the microfluidic CD result is compared with the result of a Bradford assay that was performed traditionally using microwell plate and the same samples. 

Figure 8d presents the micro-plate reader results for the Bradford assay on the microplate and the microfluidic CD. The results show that the intensity at the wavelength corresponding to the blue color increases when the protein concentration increases. The results also prove that the assay can be performed on the microfluidic CD with an accuracy very close to that of the traditional method. Therefore, multiple samples can be processed together in parallel by designing a multi-metering CD (up to 30 samples per CD). Moreover, using the resulting curve as a standard reference for the assay on the CD and a simple first order equation, the CD can be used to test the protein concentration of any unknown sample via the same procedure. Therefore, the CD platform can be utilized as a platform for detecting different diseases; for example, a liver functionality test can be performed by measuring the albumin concentration in the urine. This capability can be important for handling samples from patients with infectious diseases, such as HIV. Finally, the experiment shows that the proposed valve provides control of the burst frequency when a liquid other than water (different in terms of biochemical properties) is injected into the source chamber. 

## 5. Conclusions

In this study, an easy-to-implement passive liquid valve (PLV) for the microfluidic CD is presented. The main advantage of the proposed valve is that the valve can be actuated without an external force or trigger. In contrast to our previous work “wax valve” by Al-Faqheri *et al*. [19], the PLV valve can easily be introduced into any microfluidic process by simply injecting liquid into the venting chamber. Moreover, the lack of contact between the sample and valving material allows for the use of a variety of valving liquids without affecting the microfluidic process. Different parameters, such as the liquid height, liquid density, and venting chamber distance from the CD center, can be adjusted to control the effectiveness of the PLV valve. In addition, the ability to reverse the valve status from normally opened to normally closed and *vice versa* is a very important feature for the use of the PLV valve in multistep processes. 

The experimental and theoretical results show that the developed valve can be utilized to control the liquid burst frequency within a specific range of spinning speeds and liquid volumes. This range is defined by the ability of the utilized trapped air to withstand a specific amount of compression or expansion. Within this range, the burst frequency can be easily controlled by adjusting the height and/or density of the liquid in the venting chamber and by adjusting the position of the venting chamber with respect to the rotational center. The results show that using the proposed valve, the burst frequency of the source chamber liquid can be increased from 275 RPM to 1000 RPM.

As a proof of concept, the presented new valve is successfully utilized to demonstrate two microfluidic processes: liquid switching and liquid metering. Moreover, the Bradford assay for measuring protein concentration is performed and evaluated. In contrast to the previously presented valves, the PLV valve can reduce the spinning speed required to perform liquid switch and metering processes. Finally, the developed PLV can be integrated into biomedical assays without affecting the final results (biocompatible valve).

## Supplementary Files

Supplementary File 1
