# Supplementary material for: Development of a Passive Liquid Valve (PLV) Utilizing a Pressure Equilibrium Phenomenon on the Centrifugal Microfluidic Platform
Source: Sensors (Basel). 2015 Feb 25;15(3):4658–76. doi: 10.3390/s150304658 (PMC4435176; doi:10.3390/s150304658)
Supplement: Supplementary File 1 [file sensors-15-04658-s001.pdf]

OPEN ACCESS

sensors

ISSN 1424-8220
www.mdpi.com/journal/sensors

Supplementary Information

Development of Passive Liquid Valve (PLV) Utilizing a Pressure Equilibrium Phenomenon on the Centrifugal Microfluidic Platform. *Sensors* 2015, *15*, 4658-4676

Wisam Al-Faqheri [1,2], Fatimah Ibrahim [1,2,*], Tzer Hwai Gilbert Thio [1,2,3], Norulain Bahari [1,2], Hamzah Arof [1,2,4], Hussin A. Rothan [7], Rohana Yusof [7], Marc Madou [1,2,5,6]

1 Centre for Innovation in Medical Engineering (CIME), Faculty of Engineering, University of Malaya, 50603 Kuala Lumpur, Malaysia; E-Mails: wisamfakhri83@yahoo.com (W.A.-F.); ainbahari@yahoo.com (N.B.)

2 Department of Biomedical Engineering, Faculty of Engineering, University of Malaya, 50603 Kuala Lumpur, Malaysia

3 Faculty of Science, Technology, Engineering and Mathematics, INTI International University, Persiaran Perdana BBN, Putra Nilai, 71800 Nilai, Negeri Sembilan, Malaysia; E-Mail: gilbert_thio@hotmail.com

4 Department of Electrical Engineering, Faculty of Engineering, University of Malaya, 50603 Kuala Lumpur, Malaysia; E-Mail: ahamzah@um.edu.my

5 Department of Biomedical Engineering, University of California, Irvine, 92697 CA, USA; E-Mail: mmadou@uci.edu

6 Department of Mechanical and Aerospace Engineering, University of California, Irvine, 92697 CA, USA

7 Department of Molecular Medicine, Faculty of Medicine, University of Malaya, 50603 Kuala Lumpur, Malaysia; E-Mails: rothan@um.edu.my (H.A.R.); rohana@um.edu.my (R.Y.)

* Author to whom correspondence should be addressed; E-Mail: fatimah@um.edu.my; Tel.: + 603-7967-6818, Fax: +603-7967-6878.

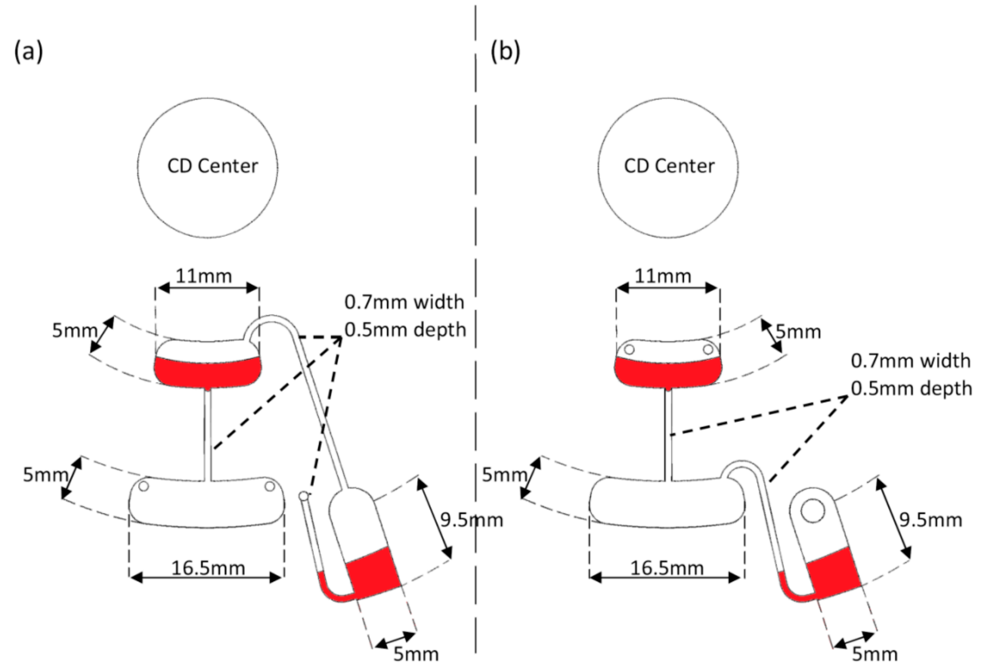

Figure S1. (**a**) and (**b**) respectively shows the designs specification of S-PLV and D-PLV. As mentioned in the main manuscript, both designs are consists of three main chambers: source chamber, destination chamber and venting chamber. The three chambers are connected together with liquid channels and venting channels. *Source chamber*: 5 mm height, 11 mm width, 1 mm depth; *Destination chamber*: 5 mm height, 16.5 mm width, 1 mm depth; *Venting chamber*: 9.5 mm height, 5 mm width, 2.5 mm depth; *Venting and liquid channels*: all 0.7 mm width and 0.5 mm depth.

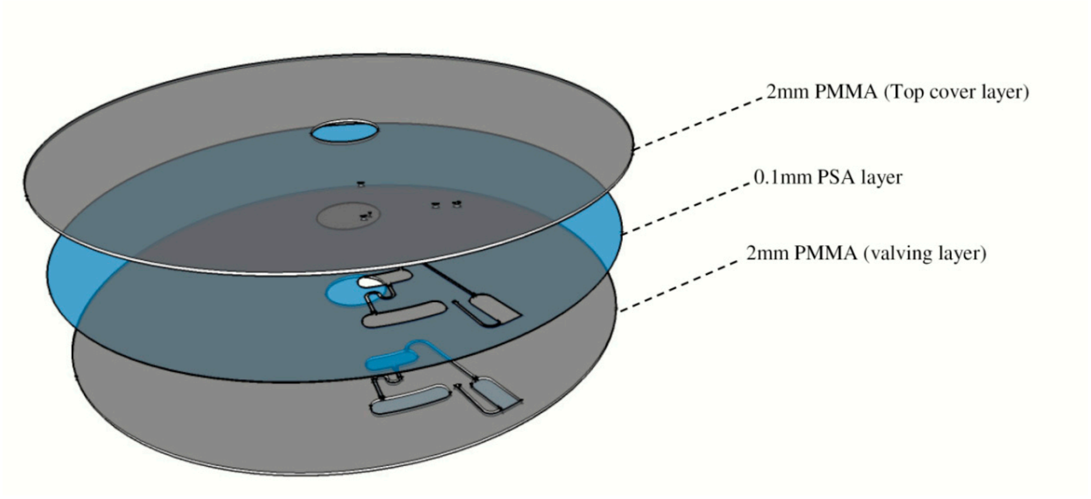

Figure S2. The microfluidic CD fabricated for this experiment consists of three layers: two PMMA layers and one PSA layer. The main microfluidic design presented in Figure S1 is engraved in the bottom 4mm PMMA layer. The Top PMMA layers acts like a cover with only venting and alignment holes are drilled through. Finally, 0.1 mm PSA adhesive layer is utilized to bind the two PMMA layers together.

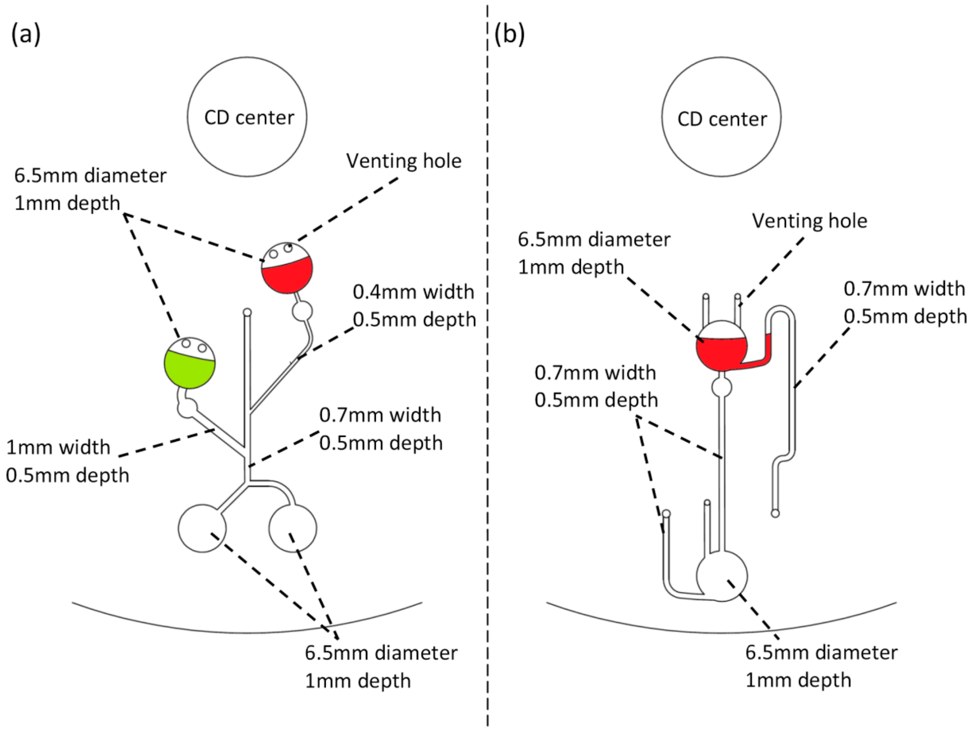

Figure S3. Dimensions details for the liquid switching experiment. *Switching layer*: source chambers and destination chambers are 6.5 mm diameter and 1mm depth. Source chamber A liquid channel is 1 mm width and 0.5 mm depth. Source chamber B liquid channel is 0.4 mm width by 0.5 mm depth. All the other channels are 0.7 mm width by 0.5 mm depth. *Venting layer:* Venting chamber A and B are 6.5 mm diameter and 1 mm depth. All the liquid and venting channels are 0.7 mm width and 0.5 mm depth.

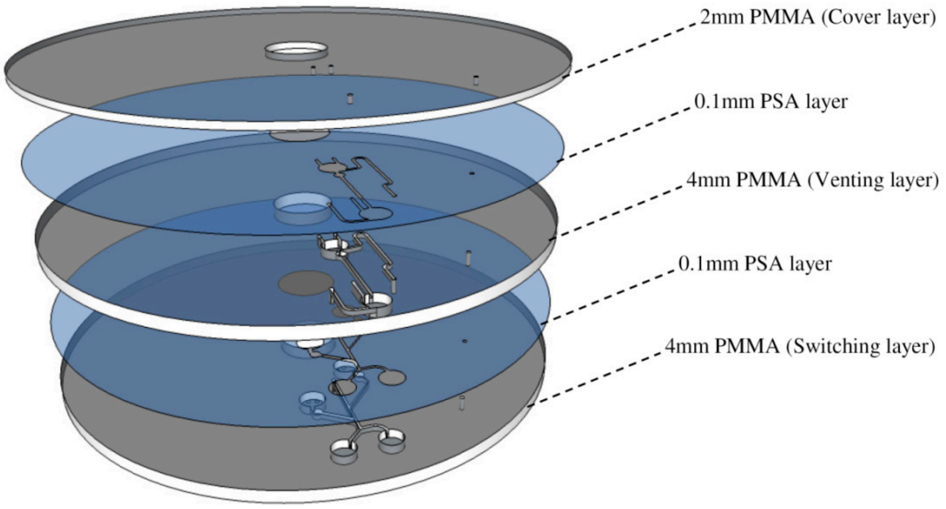

Figure S4. Liquid switching microfluidic CD layers. *Top layer:* 2 mm PMMA with only venting holes cut-through (cover layer). *Second layer:* 0.1 PSA with the venting layer design cut-through. *Third layer:* 4 mm PMMA where the venting design in Figure S3b engraved-in (venting layer). *Fourth layer:* 0.1 PSA adhesive layer with the switching design cut-through. *Fifth layer:* 4 mm PMMA layer where the switching design in Figure S3a engraved-in.

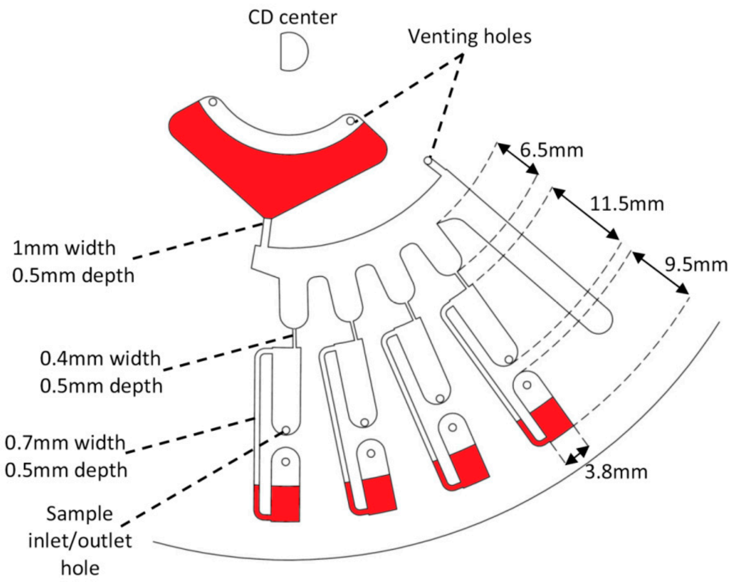

Figure S5. Liquid metering microfluidic CD design. **Chambers:** *Metering chambers*: 3.8 mm width, 6.5 mm length, and 4 mm depth; *Destination chambers:* 3.8 mm width, 11.5 mm length, and 4 mm depth; *Venting chambers*: 3.8mm width, 9.5 mm length, and 2.5 mm depth; **Channels:** *Source chamber liquid channel:* 1 mm width and 0.5 mm depth; *Metering chambers channel to the destination chambers*: 0.4 mm width and 0.5 mm depth; *Venting channels:* 0.7 mm width and 0.5 mm depth.

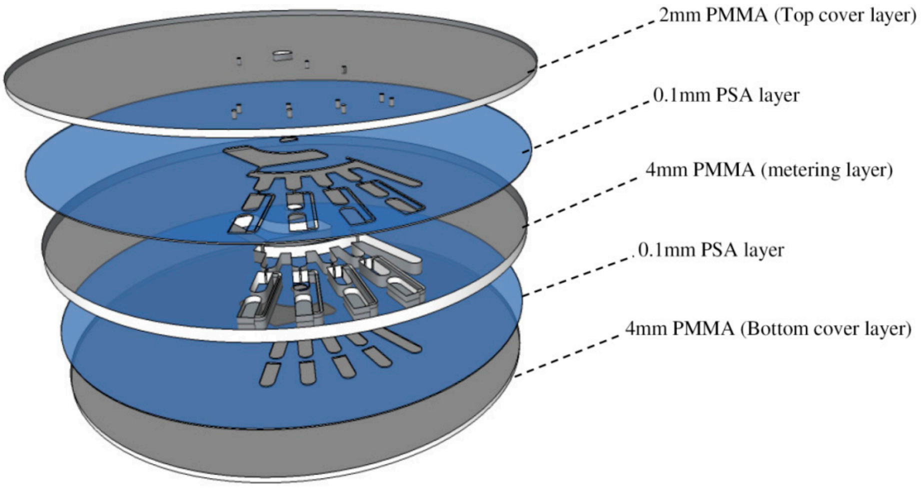

Figure S6 Liquid metering microfluidic CD layers. *Top layer:* 2 mm PMMA with only venting holes cut-through (cover layer). *Second layer:* 0.1 PSA with the metering design cut-through. *Third layer:* 4 mm PMMA where the venting design in Figure 5 engraved-in (venting layer). *Fourth layer:* 0.1 PSA adhesive layer with the metering design cut-through. *Fifth layer:* 2 mm PMMA layer (bottom cover layer).

© 2015 by the authors; licensee MDPI, Basel, Switzerland. This article is an open access article distributed under the terms and conditions of the Creative Commons Attribution license (http://creativecommons.org/licenses/by/4.0/).
